# A Phenome-Wide Mendelian Randomization and Colocalization Study Reveals Genetic Association Between PBC and Other Autoimmune Disorders

**DOI:** 10.1155/cjgh/1716853

**Published:** 2025-07-20

**Authors:** Shuyi Shi, Minghui Liu, Haonan Gao, Fang Liu, Yuhu Song

**Affiliations:** ^1^Department of Gastroenterology, Union Hospital, Tongji Medical College, Huazhong University of Science and Technology, Wuhan 430022, China; ^2^Institute of Hematology, Union Hospital, Tongji Medical College, Huazhong University of Science and Technology, Wuhan 430022, China

**Keywords:** colocalization analysis, hypothyroidism, phenome-wide association study, primary biliary cholangitis

## Abstract

**Background:** Primary biliary cholangitis (PBC) is a chronic autoimmune liver disease that is commonly associated with various other autoimmune disorders. We conducted a phenome-wide association study Mendelian randomization (MR-PheWAS) to determine genetic association between PBC and other diseases, particularly autoimmune disorders.

**Methods:** We performed a PheWAS to investigate the causal associations between PBC and related traits by conducting enrichment analysis of 35 PBC risk loci identified by prior GWAS and their matched control SNP sets in UK Biobank database. MR-PheWAS and bidirectional two-sample Mendelian randomization analysis were conducted to determine causal association between PBC and hypothyroidism. Colocalization analysis was conducted to investigate common genetic variants with hypothyroidism.

**Results:** Genetic liability to PBC was associated with a higher risk of 25 traits (hypothyroidism, asthma, allergic rhinitis, psoriasis, ulcerative colitis, and multiple sclerosis). After false discovery rate (FDR) correction, there exist 9 traits significantly difference. MR-PheWAS analysis demonstrated causal association between PBC and hypothyroidism, and bidirectional two-sample Mendelian randomization analysis was performed to validate it. The OR of hypothyroidism on PBC was 113.61(*p*=9.30E − 05), and PBC was also causally associated with hypothyroidism (OR: 1.005; *p*=4.33E − 09). Among the genes identified, CCDC88B and MMEL1 were found to have positive associations with the risk of hypothyroidism (CCDC88B: OR = 1.004, *p*=4.69E − 07; MMEL1: OR = 1.004, *p*=6.65E − 06) and FinnGen cohorts (CCDC88B: OR = 1.044; MMEL1: OR = 1.038). The two genes may be the drug targets for hypothyroidism (CCDC88B: coloc.abf-PPH4 = 94.7%; MMEL1: coloc.abf-PPH4 = 91.8%).

**Conclusions:** Our study revealed genetic association between PBC and hypothyroidism through a phenome-wide Mendelian randomization, and then, colocalization identified two potential drug targets for hypothyroidism.

## 1. Background

Primary biliary cholangitis (PBC; formerly known as primary biliary cirrhosis) is an autoimmune liver disorder characterized by the progressive destruction of intrahepatic bile ducts, leading to cholestasis, liver cirrhosis, and liver failure [1, 2]. Clinically, patients with PBC are associated with other autoimmune disorders (Hashimoto's thyroiditis; psoriasis, rheumatoid arthritis, scleroderma, Sjogren's syndrome, systemic lupus erythematosus, inflammatory bowel disease (IBD), etc). Mendelian randomization (MR) uses genetic variants to assess whether a risk factor (exposure) causally affects a disease (outcome) in a nonexperimental setting [3]. Its validity depends on three key assumptions: the genetic variants are strongly associated with the exposure; the genetic variants are not associated with outcome; the genetic variants affect the outcome only through the exposure [4–6]. Several studies revealed causal relationships between PBC and other autoimmune conditions through MR [7–12]. Phenome-wide association study (PheWAS), also known as reverse genome-wide association study (GWAS), has been increasingly used to identify novel genetic associations across a wide spectrum of phenotypes [13]. The MR-PheWAS analysis has been developed to identify the association between a set of genetic variants and a set of phenotypic variables by integrating the PheWAS and MR method. In our study, we analyzed the association between PBC and a comprehensive range of phenotypes by extracting the risk loci for PBC using PheWAS analysis. Then, MR-PheWAS and bidirectional two-sample MR analysis were performed to determine genetic association between PBC and hypothyroidism. Finally, further study revealed CCDC88B and MMEL1 share a causal variant with hypothyroidism using MR and colocalization.

## 2. Materials and Methods

### 2.1. Study Design

**Figure 1:**
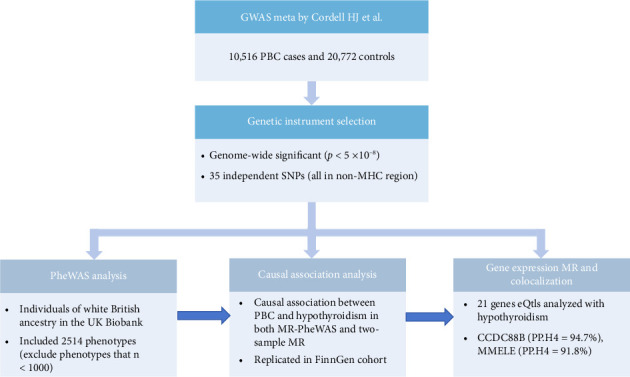
Schematic representation of the study design. Notes: GWAS, genome-wide association study; PBC, primary biliary cholangitis; PheWAS, phenome-wide association study; SNPs, single nucleotide polymorphisms.

Study design is shown in Figure 1. PheWAS analysis was performed to explore 2514 traits associated with genetic liability to PBC in the UK Biobank database. MR-PheWAS analysis and bidirectional two-sample MR were performed to determine causal association between PBC and hypothyroidism. Colocalization analysis was performed to determine the association between genes corresponding to PBC-related risk SNPs and hypothyroidism.

### 2.2. Selection of Risk Loci for PBC

There are international studies which identified potential risk loci for PBC [14], and the findings have been replicated in different articles. A recent study summarized previous findings and new risk loci [14]. The study contained five European cohorts and two Asian cohorts with a total of 10,516 cases and 20,772 control groups. We only extracted the data from the European cohorts (minor allele frequency (MAF) ≥ 0.01) due to genetic heterogeneity between European population and Asian population. We extracted the statistically significant risk loci (*p* < 5E − 08) and estimated linkage disequilibrium (LD) among these SNPs based on the 1000 Genomes European reference panel in the study [15]. SNPs in LD (*r*^2^ ≥ 0.001) were excluded [16]. Ultimately, 35 independent risk loci were selected (Table S1).

### 2.3. Control SNP Set

We compiled a SNP set to serve as controls for PheWAS analysis. A set of unlinked control SNPs (1000 Genomes Project) were generated using SNPsnap (Broad Institute) [16, 17]. According to the matching principle, four control SNPs were matched to PBC risk SNPs [18]. 140 control SNPs were matched to the 35 PBC risk SNPs on MAF (±5%), surrounding gene density (±50%), the distance to nearest gene (±50%), and, as a proxy for haplotype block size, the number of other SNPs in LD at *r*^2^ ≥ 0.50 (±50%). Therefore, a total of 175 SNPs containing 35 PBC risk SNPs and 140 SNP controls were used for the PheWAS analysis (Table S2).

### 2.4. PheWAS Analysis

UK Biobank is a population-based health research resource consisting of approximately 500,000 people, aged between the age of 38 and 73 years, who were recruited between 2006 and 2010 from the United Kingdom [19]. Detailed information on their derivation (source, original questionnaire, or measurement) is available on the UK Biobank website (https://biobank.ndph.ox.ac.uk/showcase/search.cgi). Currently, UK Biobank summary statistics were integrated in the MRC Integrative Epidemiology Unit (IEU) Open GWAS database, which include 3948 phenotypes. Finally, a total of 2514 phenotypes were used in the analysis by excluding phenotypes whose sample sizes are less than 1000 due to their low statistical power.

Then, we performed PheWAS analysis to determine the association between PBC risk loci and the 2514 phenotypes in the UK Biobank. Nominally significant SNP-trait associations (*p* < 0.01) were carried forward to trait-enrichment analysis [14]. The traits which were not completely associated with the 35 PBC risk SNPs were removed, and we used Fisher's exact tests to assess whether the traits were significantly enriched in the group of PBC risk variants using the R Statistical Programming Environment (http://www.R-project.org/version4.3.2). Fisher's exact *p*-values for trait enrichment underwent FDR correction to adjust for multiple testing.

### 2.5. MR-PheWAS Analysis

We performed MR-PheWAS analysis (IVW-RE model) using 35 PBC risk loci with 2514 traits in UK Biobank to assess the causal association between PBC and enrichment analysis results. For SNP-trait association with significantly statistical difference through MR-PheWAS (*p* < 0.05 after FDR correction), we performed bidirectional two-sample MR analyses for replication.

### 2.6. GWAS for Bidirectional MR

After FDR correction, 6 traits (hypothyroidism and levothyroxine sodium) with statistically significant differences were found in MR-PheWAS. Unfortunately, only hypothyroidism/myxoedema aligns well with our research objectives, which aims to identify the association between PBC and other autoimmune diseases. SNPs associated with hypothyroidism at the genome-wide significant level (*p* ≤ 1E − 05) were obtained from IEU Open GWAS (https://gwas.mrcieu.ac.uk) and replicated in FinnGen database. LD was determined among these SNPs based on the 1000 Genomes European reference panel [15]. SNPs in LD (*r*^2^ ≥ 0.1) with MAF < 0.01 were excluded.

### 2.7. MR Analysis

Bidirectional two-sample MR was performed to assess causal relationship between the exposure and the outcome using R package “Mendelian randomization.” Using summary statistics of SNP exposure (i.e., hypothyroidism) and SNP outcome (i.e., PBC), we used the (1) inverse-variance weighted (IVW), (2) MR-Egger, and (3) weighted median (WM) methods to test for the bidirectional causal relationship between hypothyroidism and PBC. The IVW method under the multiplicative random effects was used as the primary analysis to estimate the associations between PBC and hypothyroidism.

### 2.8. MR and Colocalization Analysis

To confirm genetic connection between PBC and hypothyroidism, we performed MR and colocalization analysis of the gene whole blood expression (expression quantitative trait locus, eQtl) corresponding to PBC risk SNPs with hypothyroidism. We characterized PBC risk SNPs using HaploReg and NCBI (https://www.ncbi.nlm.nih.gov) to annotate chromatin state and regulatory motifs surrounding each SNP [20] and found the genes corresponding to risk loci (Table S1). GWAS data for hypothyroidism were also obtained from UK Biobank. Data on gene expression were found in blood samples from the eQTLGen dataset [21].

SNPs in gene region ±1000 kb were used as instruments. Only eQTLs satisfying the following criteria were included (i) genome-wide significant association (*p* < 5E − 08); (ii) the location outside the major histocompatibility complex (MHC) region (chromosome 6, 26–34 Mb); (iii) independent association (LD clumping *r*^2^ < 0.1); and (iv) a cis-acting eQTL. We used colocalization method to obtain posterior probability for 5 hypotheses (H0–H4) in a Bayesian framework [22]. Coloc.abf-PPH4 > 80% of colocalization analysis (H4) strongly supports common variants involved in gene expression and disease risk. The analysis was performed using the default priors (*p*1=1E − 04, P2=1E − 04, and *p*12=1E − 04). F statistics were estimated for each eQTL signal. The analysis was performed using coloc package 5.2.3 in R 4.3.2 [23].

## 3. Results

### 3.1. PheWAS Analysis Identified 9 Clinical Outcomes Associated With PBC Risk SNPS After FDR Correction

**Figure 2:**
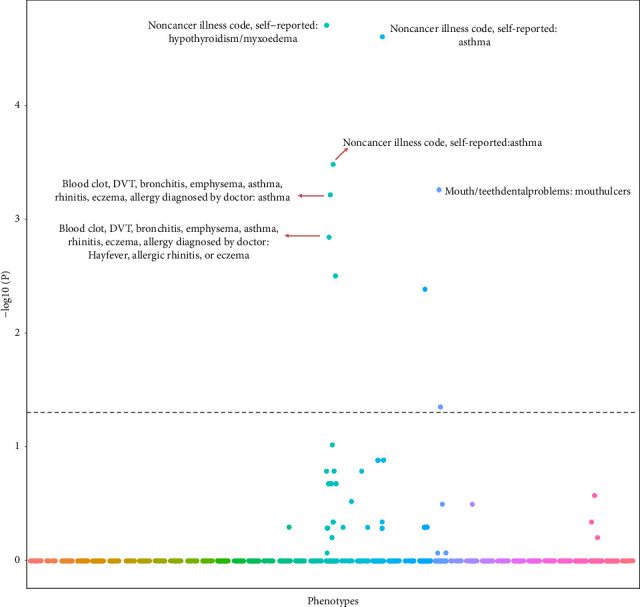
Manhattan plot for PheWAS results the horizontal axis shows the phenotypes by category, and the vertical axis shows the −log10 transformed *p* values. The dotted line indicates the corrected statistical significance level, *p*=5E − 08, and the top five statistically different traits were marked.

Using UK Biobank data, we identified 35 independent (*r*^2^ ≤ 0.001), genome-wide significant (*p* < 5E − 08) risk loci for PBC (Table S1). A SNP sets (*n* = 140) served as the controls for our PheWAS analysis (Table S2). We used the UK Biobank database to perform PheWAS analysis to identify the associations between SNPs and 2514 phenotypic traits which were associated with at least 1 PBC risk locus (*p* < 0.01). A total of 25 traits were likely to be associated with PBC SNPs compared with the controls (*p* < 0.05) (Table S3). After FDR correction, 9 traits (Table 1) were associated with PBC risk SNPs (FDR correction *p* < 0.05), and typical traits were autoimmune disorders (hypothyroidism/myxedema, asthma, and allergic rhinitis). Further study demonstrated strong association between hypothyroidism/myxedema and PBC risk SNPs. Specifically, 47.5% (16/35) of PBC risk SNPs may influence the development hypothyroidism/myxedema compared with 5.7% (8/140) of control SNPs (*p*=7.93E − 09; Table 1, Figure 2). Moreover, this association was also confirmed after FDR correction (FDR correction *p*=1.98E − 05).

### 3.2. MR-PheWAS and Bidirectional MR Demonstrated Causal Association Between Hypothyroidism and PBC.

We validated the enrichment results by performing MR-PheWAS analysis on 35 PBC risk loci. The results showed that six traits were statistically different after FDR correction (FDR correction *p* < 0.05) (Table S4, Figure S1). Hypothyroidism/myxoedema (FDR correction *p*=4.10E − 07) aligns well with our research objectives, which aims to identify the association between PBC and other autoimmune diseases. So bidirectional two-sample MR analysis was performed to validate the causal relationship between hypothyroidism and PBC.

GWAS data were obtained from OpenGWAS database (https://gwas.mrcieu.ac.uk), and then FinnGen database was used for replication. Hypothyroidism was causally associated with PBC from IVW (*p*=9.30E − 05) although no statistical differences were shown in MR-Egger (*p*=0.269) and median-weighted (*p*=0.017). The odd ratio of hypothyroidism on PBC was 113.611 (95% CI, 10.582–1219.723), but causal association of hypothyroidism on PBC was not found in FinnGen cohorts (P-IVW = 0.057).

**Figure 3:**
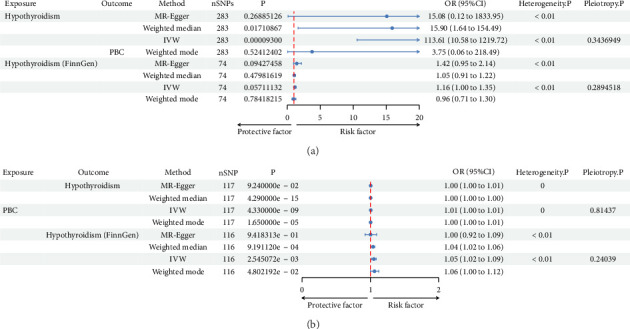
MR analysis demonstrated causal association between hypothyroidism and PBC. (a) MR analysis demonstrating causal effect of hypothyroidism on primary biliary cholangitis using data from Open GWAS (PBC: ebi-a-GCST003129, hypothyroidism: ebi-a-GCST90029022) and the FinnGen cohort (finn-b- hypothyroidism). (b) MR analysis demonstrating causal effect of primary biliary cholangitis on hypothyroidism using data from Open GWAS (PBC: ebi-a-GCST003129, hypothyroidism: ebi-a-GCST90029022) and the FinnGen cohort (finn-b- hypothyroidism).

PBC was also causally associated with hypothyroidism (OR: 1.005; 95% CI: 1.004–1.007; *p*=4.33E − 09) and replicated in FinnGen cohorts (P-IVW = 3E − 03, OR-IVW = 1.05, 95% CI of 1.02–1.09) (Table 2, Figure 3, and Figures S2–S4).

### 3.3. MR and Colocalization Analysis Demonstrated CCDC88B, MMEL1 Were Potential Targets of Hypothyroidism

We used HaploReg [20] and NCBI to search for the genes corresponding to 35 risk SNPs [14], and 21 genes were found (Table S1). MR and colocalization analysis (eQTL) were performed to identify genetic variants that affect gene expressions with hypothyroidism/myxedema using data in blood sample from the eQTL Gene dataset [21]. We identified 2 potentially causal genes with evidence of a shared genetic effect between gene expression (eQTL) and the risk of hypothyroidism. CCDC88B (rs11601860 on Chromosome 11) gene colocalized the association with hypothyroidism in whole blood (coloc.abf-PPH4 = 94.7%), and the MMEL1 (rs867436 on Chromosome 1) gene showed similar result of coloc.abf-PPH4 = 91.8%.

The two genes increased the risk of hypothyroidism in UK Biobank (CCDC88B: OR = 1.004, 95% CI = 1.003–1.006, *p*=4.69E − 07; MMEL1: OR = 1.004, 95% CI = 1.002–1.005, *p*=6.65E − 06) with IVW method (Table 3). Colocalization maps are shown in Figure S5. This suggested that the genes (CCDC88B and MMEL1) expression and hypothyroidism may share common causal variants, which could be potential drug targets for hypothyroidism.

## 4. Discussion

Clinically, PBC often occurs with other autoimmune diseases. In this study, we demonstrated that autoimmune diseases were associated with PBC using GWAS and PheWAS data. MR-PheWAS and bidirectional two-sample MR demonstrated a causal association between hypothyroidism and PBC. MR and colocalization analyses further identified CCDC88B and MMEL1 associated with PBC risk SNPs increased the risk of hypothyroidism in both UK Biobank and FinnGen cohorts.

Our study demonstrated 9 clinical outcomes enriched in the PBC risk SNPs, the majority of which are categorized as autoimmune disorders, and further study revealed causal association between PBC and hypothyroidism. In patients with PBC, there is an increased prevalence of hypothyroidism [24, 25]. Although recent study demonstrated causal effect of PBC on thyroid dysfunction using two-sample MR [8]. In our study, 9 phenotypes associated with genetic liability to PBC were determined using UK Biobank including hypothyroidism; more importantly, potential drug targets for hypothyroidism were identified. The leading cause of hypothyroidism is Hashimoto's thyroiditis, which is an autoimmune thyroiditis [26]. Autoimmune hypothyroidism is a prominent feature in PBC. Several factors contributed to causal effect of PBC on hypothyroidism. First, the change of the enzyme D3 expression result from PBC regulates the activity of thyroid hormones. Second, cholestasis caused by PBC decreases Y protein, which in turn leads to hypothyroidism [8].

We identified 2 potential causal genes with evidence of a shared genetic effect between gene expression and the risk of hypothyroidism. The SNP rs11601860 on Chromosome 11, located in the intergenic region, affects CCDC88B mRNA expression. The CCDC88B protein is abundantly expressed in immune cells, such as CD4+, CD8+ T lymphocytes, and myeloid cells. CCDC88B has pleiotropic effects on the functions of T lymphocyte, such as the maturation and activation of T lymphocyte and cytokine production [27]. Previous studies revealed abnormal expression of CCDC88B was associated with susceptibility to several autoimmune disorder, including sarcoidosis [28], IBD [29], psoriasis [30], multiple sclerosis [31], and PBC [32]. Although direct evidences on the correlation between CCDC88B and hypothyroidism have not been reported, its role in the activity and migration of immune cells suggests CCDC88B was involved in the disease. First, CCDC88B is highly expressed in immune cells and promotes the maturation and function of T cell. Its upregulation boosts the activity of T cell and dendritic cell (DC) and triggers immune attacks on thyroid tissue. Second, it also regulates the migration and activation of immune cells, potentially worsening thyroid inflammation by recruiting more immune cells, which indicates CCDC88B amplify inflammatory response. Given these, CCDC88B may be a potential therapeutic target for hypothyroidism. Our study also found genetic variant rs35776863 on Chromosome 1. The SNP-to-gene linking strategy identified MMEL1 gene associated with rs35776863. MMELI, named as Neprilysin 2(NEP2), belongs to the M13 family of metalloendopeptidases. Currently, MMELI is associated with PBC [33], rheumatoid arthritis [34], and multiple sclerosis [35, 36] Although researches do not provide direct evidence implicating the association between MMEL1 and hypothyroidism, the involvement of MMEL1 in autoimmune diseases has been reported, and there exists the overlapping gene between PBC and autoimmune thyroid disease [37]. So, it suggests that MMEL1 could be involved in the pathogenesis of hypothyroidism. In summary, we found the correlation between the expression of CCDC88B, MMEL1, and hypothyroidism risk, which indicated potential drug targets for hypothyroidism.

There are several limitations to our study. First, GWASs have contributed to our understanding of inherited susceptibility to PBC, and there remains significant missing heritability [38]. Thus, further studies should be performed to investigate genetic risk loci for PBC. Second, although our PheWAS analysis identified nine traits associated with PBC, subsequent in-depth analyses were conducted only for the association between PBC and hypothyroidism. Furthermore, while some previous MR studies have reported causal associations between PBC and IBD or irritable bowel syndrome (IBS), our results did not demonstrate significant associations with these diseases. This discrepancy could be due to differences in data sources, population characteristics, or analytical methods, highlighting the need for careful interpretation and cross-validation in future studies. Third, we identified CCDC88B and MMEL1 are potential drug targets for hypothyroidism, and experimental study and clinical trial should be performed to evaluate their therapeutic value. Finally, although sensitivity analyses such as MR-Egger and leave-one-out tests were conducted to assess the robustness of our findings, the heterogeneity still exists. To strengthen the credibility of causal inference, future studies should conduct subgroup analyses stratified by age and aim to mitigate heterogeneity arising from differences in datasets and population characteristics.

## 5. Conclusions

In our study, a phenotype-wide concept has been utilized to explore genetic association between PBC and the traits, which deepened the understanding of PBC and its comorbidities. We demonstrated causal association between PBC and hypothyroidism, and then further results revealed that CCDC88B and MMEL1 were two potential drug targets for hypothyroidism.

## Figures and Tables

**Table 1:** Traits more likely to be associated with PBC risk SNPs in PheWAS enrichment analysis.

Trait	Associated PBC SNPs	Associated control SNPs	*p*	P (FDR correction)
Noncancer illness code, self-reported: hypothyroidism/myxoedema	16/35	8/140	7.93E − 09	1.98E − 05
Treatment/medication code: levothyroxine sodium	15/35	7/140	2.00E − 08	2.50E − 05
Noncancer illness code, self-reported: asthma	11/35	3/140	3.95E − 07	0.00033
Mouth/teeth dental problems: mouth ulcers	11/35	4/140	8.82E − 07	0.00055
Blood clot, DVT, bronchitis, emphysema, asthma, rhinitis, eczema, allergy diagnosed by doctor: asthma	11/35	5/140	1.22E − 06	0.00061
Blood clot, DVT, bronchitis, emphysema, asthma, rhinitis, eczema, allergy diagnosed by doctor: hayfever, allergic rhinitis, or eczema	11/35	6/140	3.45E − 06	0.0014
Blood clot, DVT, bronchitis, emphysema, asthma, rhinitis, eczema, allergy diagnosed by doctor: none of the above	12/35	7/140	8.80E − 06	0.0031
Operation code: tonsillectomy ± adenoids	9/35	8/140	1.32E − 05	0.0041
Corneal resistance factor (right)	6/35	9/140	0.00016	0.045

*Note:* The number of PBC SNPs and control SNPs associated with each trait at *p* < 0.01 among European-ancestry individuals from UK Biobank is listed. *p* values are calculated using Fisher's exact test, comparing the frequency at which PBC SNPs and matched control SNPs were associated with each PheWAS trait.

**Table 2:** Bidirectional MR assessing causal association between primary biliary cirrhosis and hypothyroidism.

Exposure	Outcome	Method	nSNPs	*p*	OR	OR_lci95	OR_uci95	Heterogeneity P	Pleiotropy P
*A: causal association of hypothyroidism on PBC*									
Hypothyroidism	PBC	MR-Egger	283	0.269	15.083	0.124	1833.945	< 0.01	
		Weighted median	283	0.017	15.899	1.636	154.492		
		IVW	283	9.30E − 05	113.611	10.582	1219.723	< 0.01	0.314
		Weighted mode	283	0.524	3.753	0.064	218.485		

Hypothyroidism (FinnGen)	PBC	MR-Egger	74	0.094	1.423	0.946	2.141	< 0.01	
		Weighted median	74	0.480	1.053	0.912	2.151		
		IVW	74	0.057	1.158	0.996	1.347	< 0.01	0.289
		Weighted mode	74	0.784	0.959	0.709	1.296		

*B: causal association of PBC on hypothyroidism*									
PBC	Hypothyroidism	MR-Egger	117	9.24E − 02	1.005	0.999	1.010	0	
		Weighted median	117	4.29E − 15	1.004	1.003	1.005		
		IVW	117	4.33E − 09	1.005	1.004	1.007	0	0.814
		Weighted mode	117	1.65E − 05	1.004	1.002	1.005		

PBC	Hypothyroidism (FinnGen)	MR-Egger	116	0.942	1.003	0.923	1.090	< 0.01	
		Weighted median	116	0.001	1.039	1.016	1.063		
		IVW	116	0.003	1.051	1.018	1.085	< 0.01	0.240
		Weighted mode	116	0.048	1.060	1.001	1.012		

*Note:* Bidirectional MR between hypothyroidism and primary biliary cirrhosis from multiple Mendelian randomization methods (MR-Egger, weighted median, inverse-variance weighted, and weighted mode), replication in FinnGen cohort. nSNPs: the number of SNPs tested. 95% Cl: 95% confidence interval. Heterogeneity P: heterogeneity test *p* value. Pleiotropy P: pleiotropy test (MR-Egger) *p* value. A. The causal effect estimates of hypothyroidism on PBC. B. The causal effect estimates of primary biliary cirrhosis on hypothyroidism.

Abbreviation: OR: odd ration.

**Table 3:** Shared genetic variants between PBC and hypothyroidism through MR and colocalization analysis.

Gene	SNP	Region	Outcome	Method	nSNPs	*p*	OR	PP.H4.abf (%)
CDC88B	rs11601860	chr2:64107676-64125006	Hypothyroidism	IVW	41	4.69E − 07	1.0043	94.7
MMEL1	rs867436	chr1:2522078–2564455	Hypothyroidism	IVW	22	6.65E − 06	1.0035	91.8

*Note:* Region: genomic region including chromosome and the start and stop region. IVW: inverse-variance weighted method. nSNPs: the number of SNPs tested in the genomic region. P: inverse-variance weighted method *P* value. PP.H4.abf: the posterior probability of hypothyroidism and PBC sharing a causal variant.

Abbreviation: OR: odd ration.

## Data Availability

The data that support the findings of this study are available from the corresponding author upon reasonable request.

## Nomenclature

PBC: Primary biliary cholangitis
IBD: Inflammatory bowel disease
PheWAS: Phenome-wide association study
MR: Mendelian randomization
GWAS: Genome-wide association studies
MR-PheWAS: Phenome-wide Mendelian randomization
SNP: Single nucleotide polymorphism
IVW: Inverse-variance weighted
WM: Weighted median
eQtl: Expression quantitative trait locus
MHC: Major histocompatibility complex
LD: Linkage disequilibrium
FDR: False discovery rate

## Supporting Information

Table S1: Genetic variants for PBC in prior GWAS and included in our PheWAS analysis. Table S2: PBC risk SNPs and 4:1 matched control SNP set. Table S3: Traits are associated with PBC risk SNPs in PheWAS enrichment analysis (*P* < 0.05). Table S4: MR-PheWAS results demonstrated causal association between hypothyroidism and PBC (P-FDR< 0.05). Figure S1: Manhattan plot of MR-PheWAS for the primary biliary cirrhosis risk SNPs with all phenotypes included by category. Figure S2: Scatter plots for the causal association between PBC and hypothyroidism. Figure S3: Funnel plots of MR estimation between PBC and hypothyroidism. Figure S4: Results of “leave-one-out” sensitivity analysis of MR estimation between PBC and hypothyroidism. Figure S5: Colocalization maps of CCDC88B and MMEL1 for hypothyroidism.
