# Supplementary material for: A Phenome-Wide Mendelian Randomization and Colocalization Study Reveals Genetic Association Between PBC and Other Autoimmune Disorders
Source: Can J Gastroenterol Hepatol. 2025 Jul 20;2025:1716853. doi: 10.1155/cjgh/1716853 (PMC12301091; doi:10.1155/cjgh/1716853)
Supplement: Supporting Information — Additional supporting information can be found online in the Supporting Information section. [file 1716853.f1.docx]

**Supplementary Data**

**A phenome-wide** **Mendelian randomization and colocalization study reveals genetic association between PBC and other autoimmune disorders**

Shuyi Shi^1^, Minghui Liu^1^, Haonan Gao^1^, Yuhu Song^1^

^1^Department of Gastroenterology, Union Hospital, Tongji Medical College, Huazhong University of Science and Technology, Wuhan 430022, China

***Corresponding author:***

Yuhu Song and Fang Liu

Fang Liu^2^

Institute of Hematology, Union Hospital, Tongji Medical College, Huazhong University of Science and Technology, Wuhan 430022, China

Email: fangliuwh@163.com; Telephone: 0086-27-85726005.

Yuhu Song^1^

Department of Gastroenterology, Union Hospital, Tongji Medical College, Huazhong University of Science and Technology; Wuhan 430022, China

Email: yuhusong@163.com

**TableS1: Genetic variants for PBC in prior GWAS and included in our PheWAS analysis**

| SNP | EA | OA | Pos | Chr | P | Gene | Function |  |
| --- | --- | --- | --- | --- | --- | --- | --- | --- |
| rs11601860 | T | A | 64110422 | 11 | 1.45E-10 | CCDC88B | intronic |  |
| rs867436 | T | C | 2523723 | 1 | 2.99E-09 | MMEL1 | intronic |  |
| rs6679356 | C | T | 67820194 | 1 | 6.61E-63 | IL12RB2 | intronic |  |
| rs10802191 | A | T | 117065083 | 1 | 2.81E-08 | CD58 | intronic |  |
| rs945635 | G | C | 157670290 | 1 | 1.59E-08 | FCRL3 | 5’-UTR |  |
| rs55734382 | T | C | 201019059 | 1 | 2.06E-09 | CACNA1S | intronic |  |
| rs34655300 | T | C | 25514333 | 2 | 5.23E-10 | DNMT3A | intronic |  |
| rs859767 | G | A | 135341200 | 2 | 1.54E-09 | TMEM163 | intronic |  |
| rs3771317 | C | T | 191543662 | 2 | 2.40E-22 | NAB1 | intronic |  |
| rs6550965 | A | C | 25383587 | 3 | 3.65E-14 | RARB | intronic |  |
| rs2293370 | A | G | 119219934 | 3 | 5.54E-25 | TIMMDC1 | intronic |  |
| rs7805218 | A | G | 20378801 | 7 | 4.12E-08 | ITGB8 | intronic |  |
| rs60600003 | G | T | 37382465 | 7 | 4.70E-13 | ELMO1 | intronic |  |
| rs7097397 | A | G | 50025396 | 10 | 2.42E-10 | WDFY4 | missense |  |
| rs124419634 | G | C | 111239365 | 11 | 8.28E-09 | POU2AF1 | intronic |  |
| rs1800693 | C | T | 6440009 | 12 | 1.19E-16 | TNFRSF1A | intronic |  |
| rs3784099 | A | T | 68749927 | 14 | 8.31E-17 | RAD51B | intronic |  |
| rs72699866 | A | G | 93114787 | 14 | 2.89E-11 | RIN3 | intronic |  |
| rs9652601 | A | G | 11174365 | 16 | 6.69E-24 | CLEC16A | intronic |  |
| rs2304256 | A | C | 10475652 | 19 | 4.43E-17 | TYK2 | missense |  |
| rs3745516 | A | G | 50926742 | 19 | 2.65E-30 | SPIB | intronic |  |
| rs79577483 | G | A | 68036939 | 16 | 1.23E-11 | DUS2L | NA |  |
| rs589446 | T | G | 159733527 | 3 | 1.96E-55 | NA | NA |  |
| rs7674640 | C | T | 103540780 | 4 | 1.56E-22 | NA | NA |  |
| rs2546890 | G | A | 158759900 | 5 | 2.51E-11 | NA | NA |  |
| rs7774434 | C | T | 32657578 | 6 | 3.68E-104 | NA | NA |  |
| rs9591325 | C | T | 50811220 | 13 | 2.14E-19 | NA | NA |  |
| rs59643720 | C | A | 103564807 | 14 | 2.73E-38 | NA | NA |  |
| rs11117432 | A | G | 86019271 | 16 | 2.82E-24 | NA | NA |  |
| rs1808094 | T | C | 67526026 | 18 | 2.79E-09 | NA | NA |  |
| rs137687 | A | G | 39740078 | 22 | 2.37E-23 | NA | NA |  |
| rs12123169 | A | T | 197780966 | 1 | 9.75E-18 | NA | NA |  |
| rs2327832 | G | A | 137973068 | 6 | 2.31E-10 | NA | NA |  |
| rs9533122 | A | G | 43055002 | 13 | 5.83E-13 | NA | NA |  |
| rs1119132 | A | G | 27403469 | 16 | 6.58E-10 | NA | NA |  |

Position in GRCh37/hg19.

Minor allele frequency in European-ancestry subjects from the 1000 genomes project. EA: effect allele, OA: other allele, Pos: position, Chr: chromosome, Gene: gene corresponding to PBC risk SNPs.

NA: genes and gene’ function corresponding to PBC risk SNPs can't be find by HaploReg or NCBI.

**TableS2: PBC risk SNPs and 4:1 matched control SNP-set**

| TYPE | SNP | TYPE | SNP | TYPE | SNP | TYPE | SNP | TYPE | SNP |
| --- | --- | --- | --- | --- | --- | --- | --- | --- | --- |
| test | rs867436 | test | rs2327832 | test | rs11117432 | control | rs35573509 | control | rs2835422 |
| test | rs6679356 | test | rs7805218 | test | rs1808094 | control | rs76644959 | control | rs743728 |
| test | rs10802191 | test | rs60600003 | test | rs2304256 | control | rs6692233 | control | rs6788567 |
| test | rs945635 | test | rs7097397 | test | rs3745516 | control | rs8060188 | control | rs13410636 |
| test | rs12123169 | test | rs11601860 | test | rs137687 | control | rs6781472 | control | rs927603 |
| test | rs55734382 | test | rs12419634 | control | rs448131 | control | rs2500017 | control | rs58470105 |
| test | rs34655300 | test | rs1800693 | control | rs62114548 | control | rs13261945 | control | rs359095 |
| test | rs859767 | test | rs9533122 | control | rs138305 | control | rs3093115 | control | rs13170391 |
| test | rs3771317 | test | rs9591325 | control | rs6664662 | control | rs62434426 | control | rs112103000 |
| test | rs6550965 | test | rs3784099 | control | rs62488989 | control | rs9329255 | control | rs12503141 |
| test | rs2293370 | test | rs72699866 | control | rs62183853 | control | rs12496456 | control | rs1860849 |
| test | rs589446 | test | rs59643720 | control | rs1606292 | control | rs6425661 | control | rs58172091 |
| test | rs7674640 | test | rs9652601 | control | rs6753583 | control | rs12998294 | control | rs1748432 |
| test | rs2546890 | test | rs1119132 | control | rs59844400 | control | rs10892740 | control | rs2917964 |
| test | rs7774434 | test | rs79577483 | control | rs11650740 | control | rs9611791 | control | rs1418634 |

| TYPE | SNP | TYPE | SNP | TYPE | SNP | TYPE | SNP | TYPE | SNP |
| --- | --- | --- | --- | --- | --- | --- | --- | --- | --- |
| control | rs7739816 | control | rs196291 | control | rs112981157 | control | rs1102598 | control | rs1848710 |
| control | rs10880393 | control | rs28895005 | control | rs13254454 | control | rs4310854 | control | rs2700816 |
| control | rs12440998 | control | rs4386759 | control | rs6030766 | control | rs10742976 | control | rs17517366 |
| control | rs62439776 | control | rs923663 | control | rs1169263 | control | rs55757307 | control | rs11762322 |
| control | rs4733687 | control | rs4652369 | control | rs10470569 | control | rs9913332 | control | rs12480610 |
| control | rs6685531 | control | rs570344075 | control | rs3821277 | control | rs7594326 | control | rs6508971 |
| control | rs11688859 | control | rs35652 | control | rs2380424 | control | rs55690483 | control | rs34107354 |
| control | rs10750756 | control | rs7038361 | control | rs1870901 | control | rs7098081 | control | rs112360546 |
| control | rs80092855 | control | rs2748821 | control | rs1247140 | control | rs76181827 | control | rs17715553 |
| control | rs4785586 | control | rs9276853 | control | rs10906936 | control | rs113103441 | control | rs74171467 |
| control | rs6458973 | control | rs2404969 | control | rs11123840 | control | rs12212896 | control | rs274629 |
| control | rs1230100 | control | rs2242926 | control | rs640996 | control | rs9725131 | control | rs2638405 |
| control | rs7530137 | control | rs184973932 | control | rs2368655 | control | rs2870488 | control | rs59790257 |
| control | rs6121789 | control | rs8066195 | control | rs2756957 | control | rs1912234 | control | rs12579052 |
| control | rs738880 | control | rs11258844 | control | rs17700286 | control | rs2334096 | control | rs56994534 |
| control | rs2582419 | control | rs34345959 | control | rs6014144 | control | rs2473488 | control | rs2634198 |
| control | rs4456560 | control | rs3891956 | control | rs6442720 | control | rs6982004 | control | rs7282733 |
| control | rs34493940 | control | rs2353588 | control | rs10901774 | control | rs34354954 | control | rs12599166 |
| control | rs36026951 | control | rs11850898 | control | rs5753201 | control | rs16967863 | control | rs34281729 |
| control | rs11001096 | control | rs1188273 | control | rs593719 | control | rs200483363 | control | rs4245265 |

35 PBC risk locus SNPs matched with 140 control SNPs, for a total of 175 SNPs were used for PheWAS analysis. PBC: primary biliary cholangitis.

**TableS3: Traits are associated with PBC risk SNPs in PheWAS enrichment analysis (P<0.05)**

| Trait | Associated PBC SNPs | Associated control SNPs | P |
| --- | --- | --- | --- |
| Non-cancer illness code, self-reported: hypothyroidism/myxoedema | 16 | 8 | 7.93E-09 |
| Treatment/medication code: levothyroxine sodium | 15 | 7 | 2.00E-08 |
| Non-cancer illness code, self-reported: asthma | 11 | 3 | 3.95E-07 |
| Mouth/teeth dental problems: Mouth ulcers | 11 | 4 | 8.82E-07 |
| Blood clot, DVT, bronchitis, emphysema, asthma, rhinitis, eczema, allergy diagnosed by doctor: Asthma | 11 | 4 | 1.22E-06 |
| Blood clot, DVT, bronchitis, emphysema, asthma, rhinitis, eczema, allergy diagnosed by doctor: Hayfever, allergic rhinitis or eczema | 11 | 5 | 3.45E-06 |
| Blood clot, DVT, bronchitis, emphysema, asthma, rhinitis, eczema, allergy diagnosed by doctor: None of the above | 12 | 8 | 8.80E-06 |
| Operation code: tonsillectomy +/- adenoids | 9 | 3 | 1.32E-05 |
| Corneal resistance factor (right) | 6 | 1 | 0.00016 |
| Non-cancer illness code, self-reported: psoriasis | 7 | 3 | 0.00038 |
| Treatment/medication code: ventolin 100micrograms inhaler | 6 | 2 | 0.00057 |
| Treatment/medication code: thyroxine product | 6 | 2 | 0.00063 |
| Age asthma diagnosed | 5 | 1 | 0.00092 |
| Vitamin and mineral supplements: Folic acid or Folate (Vit B9) | 4 | 0 | 0.00089 |
| Non-cancer illness code, self-reported: ulcerative colitis | 4 | 0 | 0.00098 |
| Age hay fever, rhinitis or eczema diagnosed | 6 | 3 | 0.00145 |
| Non-cancer illness code, self-reported: multiple sclerosis | 5 | 1 | 0.00146 |
| Non-cancer illness code, self-reported: hayfever/allergic rhinitis | 6 | 3 | 0.00150 |
| Diagnoses - main ICD10: K51.9 Ulcerative colitis, unspecified | 5 | 1 | 0.0020 |
| Doctor diagnosed hayfever or allergic rhinitis | 5 | 2 | 0.0024 |
| Mouth/teeth dental problems: None of the above | 5 | 2 | 0.0028 |
| Types of physical activity in last 4 weeks: Other exercises (eg: swimming, cycling, keep fit, bowling) | 5 | 2 | 0.0028 |
| Treatment/medication code: folic acid product | 4 | 1 | 0.0046 |
| Diagnoses - secondary ICD10: J45.9 Asthma, unspecified | 4 | 1 | 0.0044 |
| Non-cancer illness code, self-reported: eczema/dermatitis | 4 | 1 | 0.0045 |

The number of PBC SNPs and control SNPs associated with each trait at p < 0.01 among European-ancestry individuals from UK Biobank are listed. P values are calculated using Fisher’s exact test, comparing the frequency at which PBC SNPs and matched control SNPs were associated with each PheWAS trait.

**TableS4: MR-PheWAS results demonstrated casual association between hypothyroidism and PBC (P-FDR< 0.05)**

| Trait | method | P | P(FDR) |
| --- | --- | --- | --- |
| Treatment/medication code: levothyroxine sodium | IVW | 1.53E-10 | 3.82E-07 |
| Non-cancer illness code, self-reported: hypothyroidism/myxoedema | IVW | 3.28E-10 | 4.10E-07 |
| Diagnoses - secondary ICD10: E03.9 Hypothyroidism, unspecified | IVW | 3.10E-07 | 2.58E-04 |
| Diagnoses - secondary ICD10: R51 Headache | IVW | 6.49E-05 | 0.040 |
| Long-standing illness, disability or infirmity | IVW | 8.07E-05 | 0.040 |
| Birth weight of first child | IVW | 1.01E-04 | 0.042 |

IVW: Inverse variance weighted.

PBC: primary biliary cholangitis.

FDR: False Discovery Rate.

**FigureS1: Manhattan plot of MR-PheWAS for the primary biliary cirrhosis risk SNPs with all phenotypes included by category**


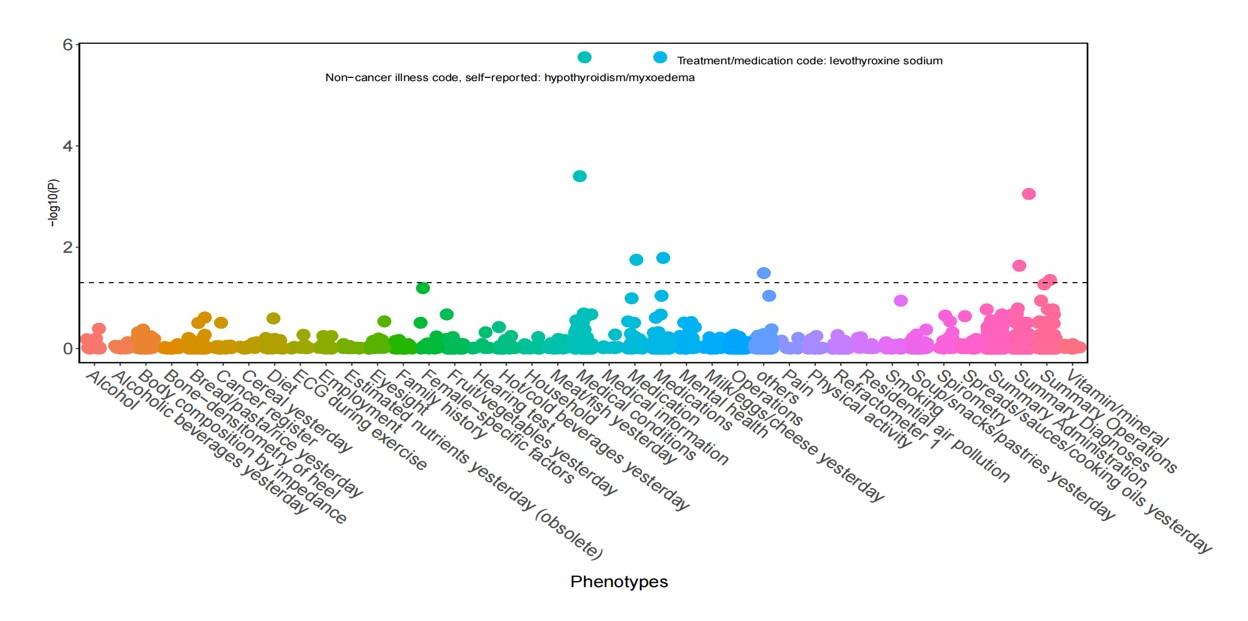


Traits were statistically different after FDR correction.

**FigureS2: Scatter plots for the causal association between PBC and hypothyroidism**


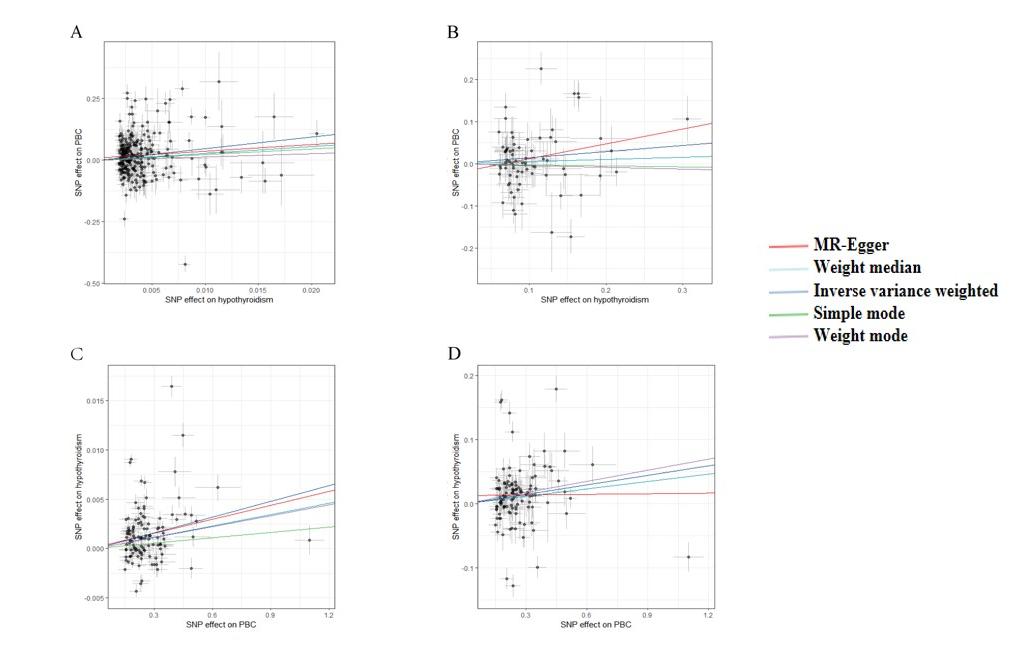


A: PBC on hypothyroidism;

B: replication practice for PBC on hypothyroidism;

C: hypothyroidism on PBC;

D: replication practice for hypothyroidism on PBC

Note: PBC: primary biliary cholangitis.

**Figure S3: Funnel plots of MR estimation between PBC and hypothyroidism**


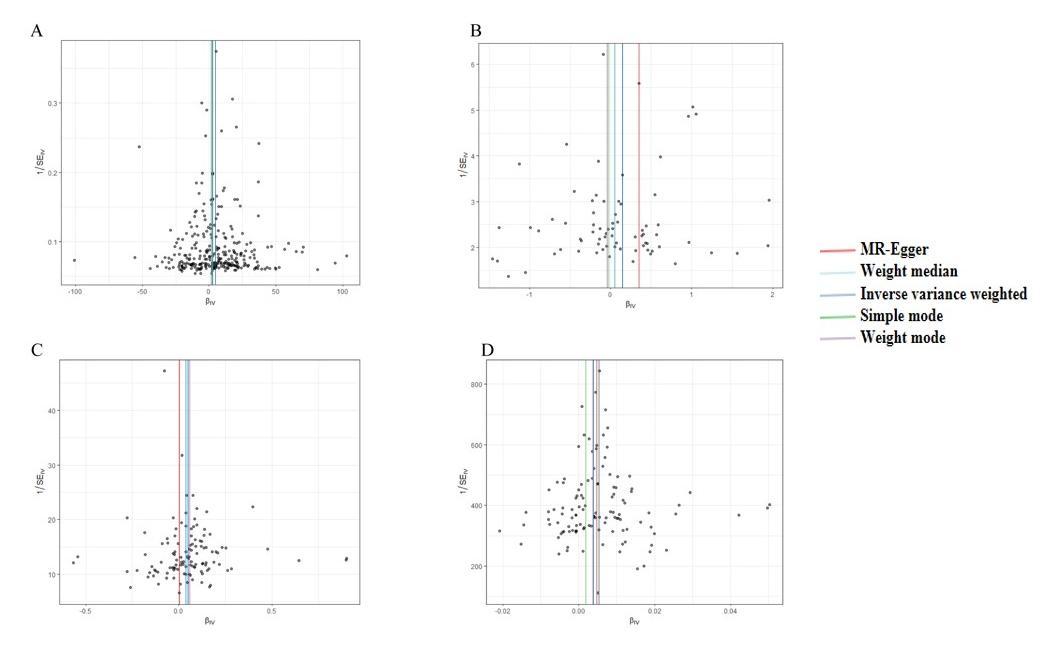


A: PBC on hypothyroidism;

B: replication practice for PBC on hypothyroidism;

C: hypothyroidism on PBC;

D: replication practice for hypothyroidism on PBC hypothyroidism

Note: PBC: primary biliary cholangitis.

**Figure S4: results of “leave-one-out” sensitivity analysis of MR estimation between PBC and hypothyroidism**


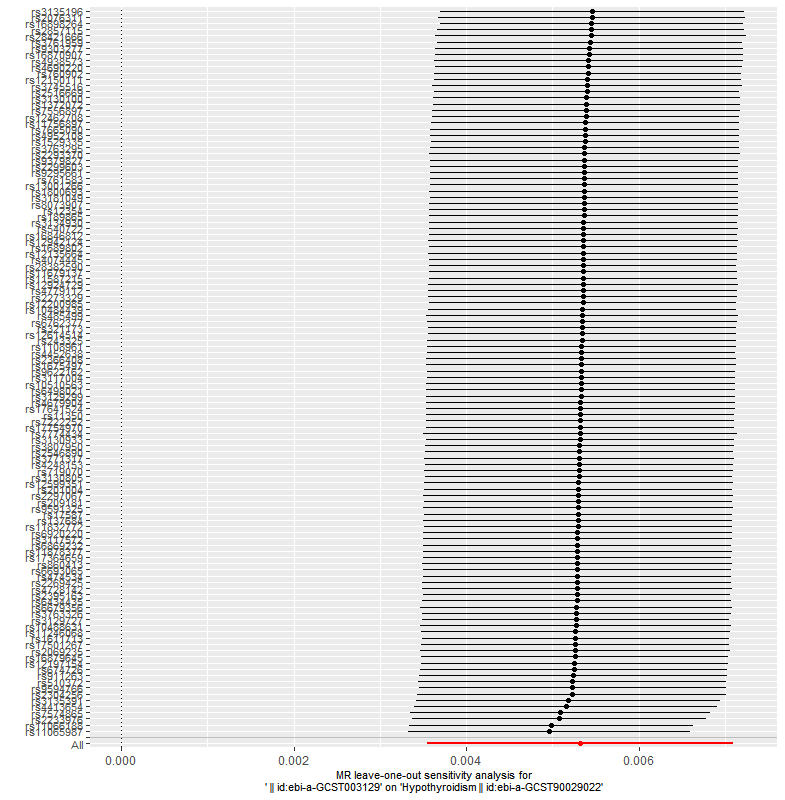


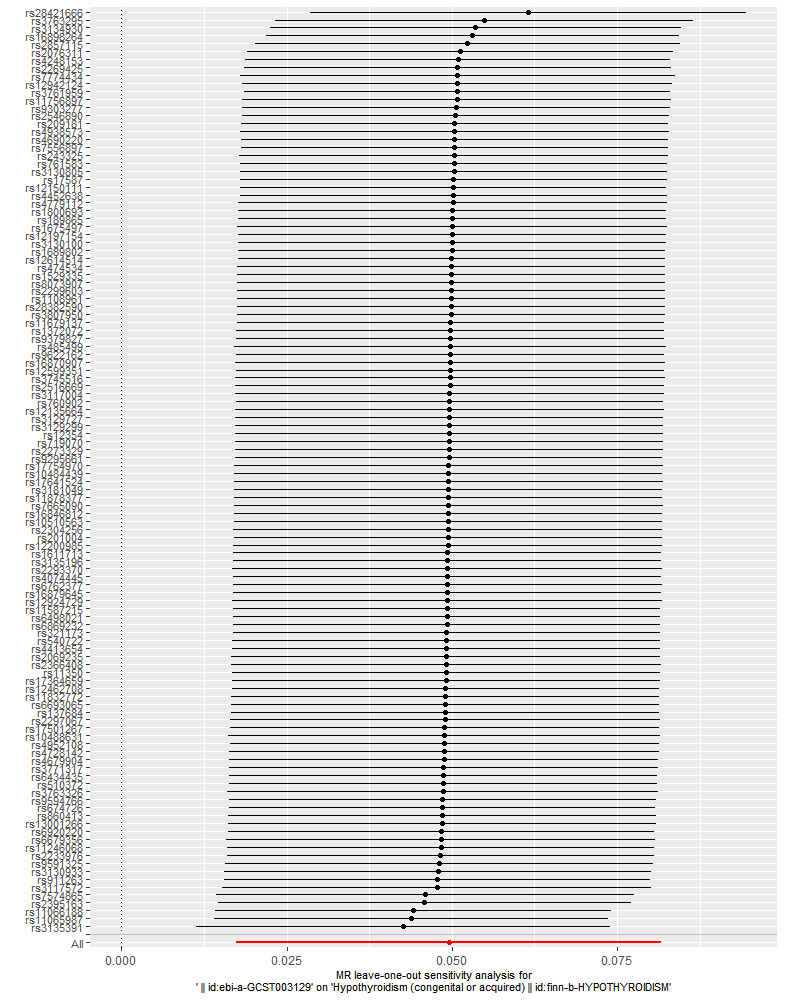


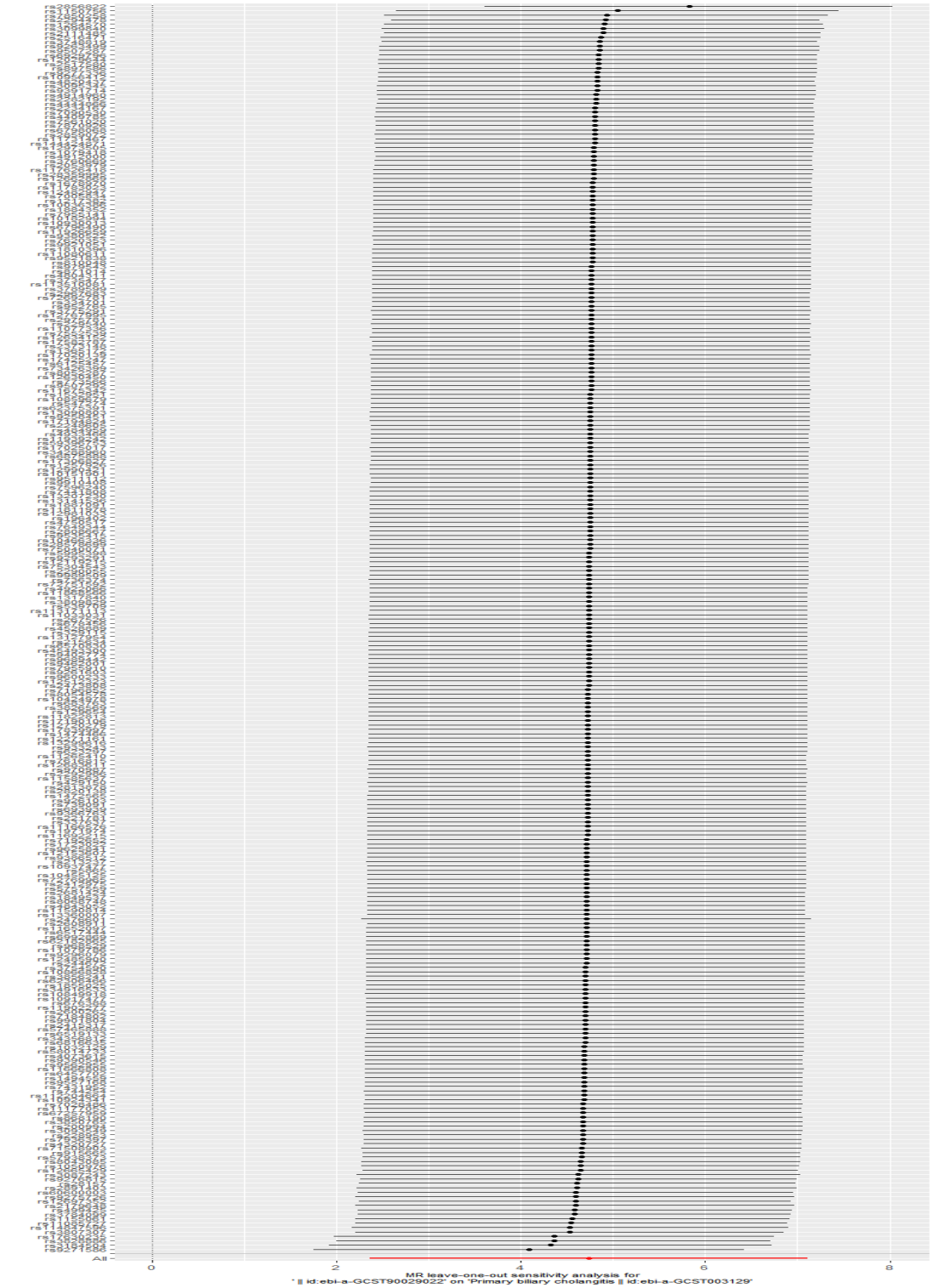


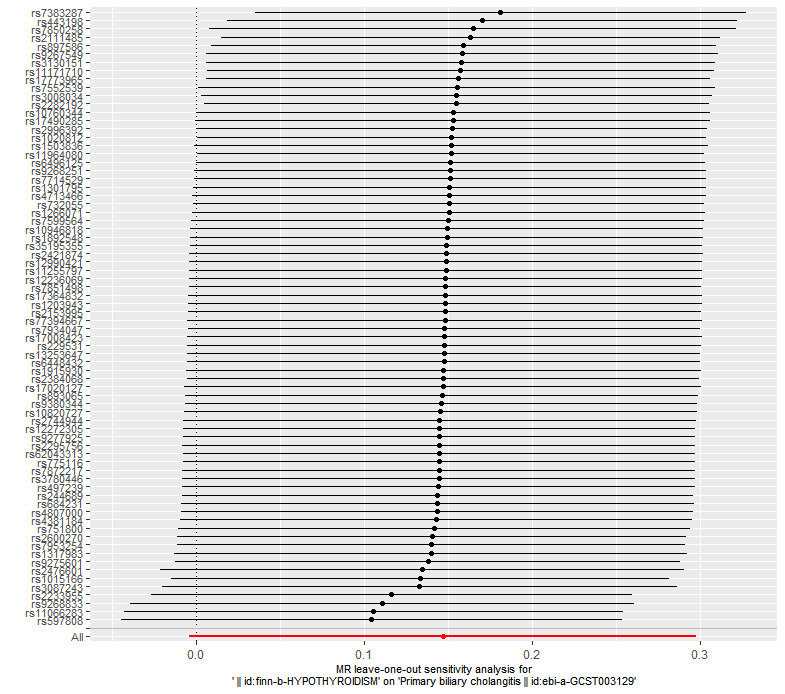


Note: PBC: primary biliary cholangitis.

**Figure S5: Co-localization maps of CCDC88B and MMEL1 for hypothyroidism**


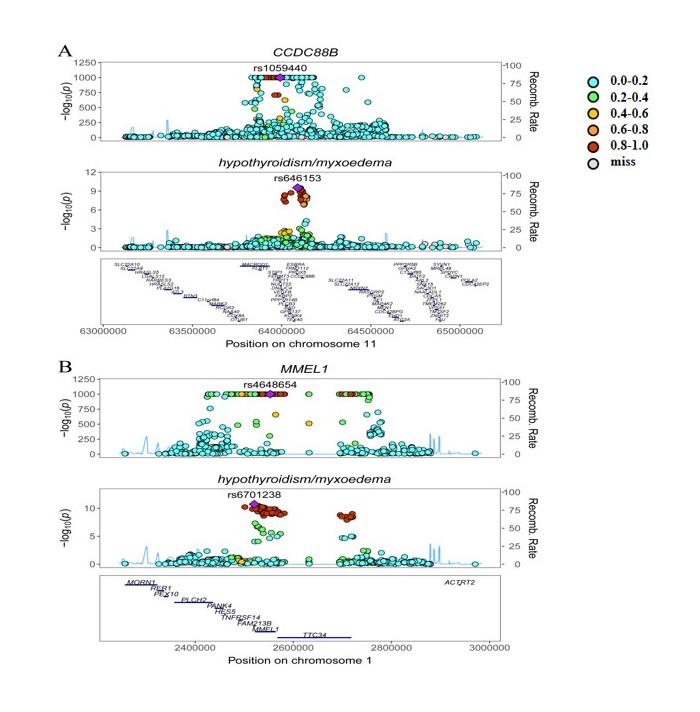


A. Co-localization maps of CCDC88B for hypothyroidism.

B. Co-localization maps of MMEL1 for hypothyroidism.
